# How do Faces Influence Behavior? A Proposal for Distinguishing between Mechanisms that Involve Cognitive inferences, Emotional Feelings, and Unconscious Affective Reactions

**DOI:** 10.1007/s42761-025-00350-9

**Published:** 2026-01-10

**Authors:** Zaiyao Zhang, Piotr Winkielman

**Affiliations:** 1 Department of Psychology, University of California, San Diego, CA USA; 2 SWPS University, Warsaw, Poland

**Keywords:** Facial expression, Subjective experience, Feelings, Affective influence, Consciousness

## Abstract

Emotional stimuli – such as facial expressions, images, or text – can influence behavior, including important decisions. This influence is complex as these stimuli may engage multiple psychological and physiological processes. The processes encompass (i) perception, attention, and memory, (ii) motor patterns, (iii) central and peripheral circuitry, (iv) subjective feelings, and (v) inferences regarding the stimulus’ meaning. All these processes can shape subsequent behavior. For example, a smile may communicate permission and encouragement to explore. A smile may also lift one’s conscious mood, which, in turn, may serve as a basis for more favorable judgments. However, other mechanisms can operate without involving conscious feelings. In fact, in some studies on facial expressions, researchers observe shifts in attention, perception, and memory, changes in physiology (e.g., amygdala activation, sweating, respiration, heart rate) and behavior (e.g., approach, consumption, risky decisions), without participants reporting any feelings. In other studies, observed changes in behavior are causally unrelated to changes in feelings. In this article, we propose a framework distinguishing informational, feeling-based, and unconscious affective pathways of affective influence. We illustrate our framework with key studies, focusing on the variety of influences by facial expressions.

An emotion stimulus, such as a facial expression (e.g., a smile, fear, disgust, or a frown), can influence behaviors via multiple mechanisms. Some mechanisms are clearly non-emotional. For example, a dinner host’s smile can make us take a sip of a new mixed drink because we read it as a sign of permission or encouragement (a thumbs-up, a hand wave, or the word “drink” would have the same effect). The stimulus (smile) might be called “emotional,” but it influences behavior via a "cold", informational pathway. However, other mechanisms involve "hot", affective components (we address the definitional issues shortly). For example, seeing our dinner host’s disgusted face may spoil our mood and stop us from trying the new drink. These affective mechanisms may involve motor processes, as when we spontaneously return the host’s smile, subtly lean towards a friend at the table, or freeze upon noticing an angry guest. Other affective mechanisms may involve perceptual and attentional biases, as when seeing our companion’s fearful face makes us look down at our own food and mistake a benign peppercorn for a bug.

The literature recognizes this variety, often defining “affect” or “emotion” as a loose aggregation of multiple components organized around valence. These include internal physiological states (e.g., changes in heart rate, perspiration, respiration, hormonal and neurotransmitter release), expressions (e.g., in the face, voice, touch, and body), action tendencies (e.g., flight, freeze, approach), appraisals of the eliciting events, and subjective feelings – that is, phenomenal experiences.

Importantly, theorists often disagree on the necessity or importance of the specific components for calling a state “affect” and “emotion” and the hypothesized relationship between components. Some propose that in “proper” emotion states these components are tightly synchronized. For example, a subjective feeling of fear can be accompanied by all the characteristic physiological states, expressions, action tendencies, and cognitive appraisals – fully deserving the term “emotion episode” (Grandjean et al., 2008). However, other theorists point out that different components vary independently and some components can be lacking even in strong emotion states (e.g., freezing, Lang & Bradley, 2010). Empirically, assessing the co-occurrence of these components is challenging, and evidence for strong synchronization remains limited (for a recent review, see Mauss et al., 2024).

Despite these considerations, some researchers propose that conscious feelings (subjective experience) are definitional for emotion (Clore et al., 1994; Frijda, 1999; Ortony, 2025). Others contend that the term “emotion” should be reserved for cognitively elaborated, and thus likely conscious processes, and use the term “affect” for basic, not necessarily conscious processes involving valence (Barrett, 2017). Some theorists avoid the term affect when discussing low-level, unconscious processes, preferring terms like “defense reactions” (LeDoux & Brown, 2017).

Importantly, most neuroscientists and animal researchers don’t assign subjective feelings an essential role. They take a functional perspective on which terms like “affect” and “emotions” simply refer to valence-related processes helping an organism behave adaptively. They point out that assessment of stimulus valence – whether it is hospitable or unhospitable – is a key task for any organism to maintain homeostasis and self-preservation (Bechtel & Bich, 2024). The determination of what is good or bad for the organism (what it “likes” or “dislikes”), mobilization and organization of physiological and processing resources, and producing appropriate behaviors such as approach or avoidance constitute the core, most fundamental function of affective and motivational processes. To quote “*emotion states evolved in order to allow us to cope with environmental challenges in a way that is more flexible*,* predictive and context-sensitive than are reflexes*,* but that doesn’t yet require the full flexibility of volitional*,* planned behavior*” (Adolphs, 2016, p. 25). This functional perspective suggests that many of these mechanisms evolved prior to those responsible for conscious emotional experience and rely on distinct neural circuits. This perspective also allows for investigation of affective mechanisms across species, including invertebrates with limited (if any) capacity for conscious feelings, such as crawfish, flies, or bees (Anderson & Adolphs, 2014; Paul et al., 2020; but see Chittka et al., 2025).

Even in humans, the dissociation between basic affective reactions and conscious feelings can be illustrated with some simple examples. A conscious experience sometimes follows, rather than precedes, a defensive reaction, such as feeling pain *after* removing a hand from a hot stove, feeling fear *after* jumping away from an approaching car, or feeling anger *after* striking an intruder (Newen & Montemayor, 2023). Some reward stimuli (e.g., drug cues for an addict) can become attention magnets and drive approach behavior, without eliciting the conscious experience of pleasure (Berridge, 2023). Finally, physiological reactions and action tendencies related to “defense responses” (e.g., cardiovascular changes, startle potentiation, sweating) can occur without the conscious experience of fear (LeDoux, 2012). In fact, drugs that reduce the subjective feeling of fear often fail to suppress basic defensive reactions, and drugs that suppress basic defensive reactions have little effect on the feeling of fear (LeDoux, 2016). Note that in all these examples, the underlying mechanisms are more complex and more context-sensitive than “mere reflexes” and should not be dismissed as such. In fact, as discussed later, they are involved in intricate influences on perception, attention, and learning (Adolphs, 2016). Furthermore, they are not just “informational”, in the sense of being primarily concerned with reflecting (representing) the content of the outside world (Zajonc, 1980). As mentioned, they are ultimately concerned with the question of valence: “is it good or bad for the organism?” Accordingly, they structure the behavior around the approach-avoidance axis, are tied to motivational processes, and tend to evoke physiological and bodily responses, thus deserving to be classified as affective. In short, for the purpose of the current paper, we adopt a broad perspective according to which it seems most useful to think of affect as a loosely integrated pattern of characteristic features organized around valence, but none of them are “definitional” or necessary (Newen et al., 2015). The term “emotion” is then used to describe more specific patterns within the broader category of valence, such as distinctions between anger, disgust, fear, sadness, etc.

The current paper draws on these insights and suggests that the distinction between informational assessments, conscious feelings, and affective reactions makes it useful to consider three general pathways through which emotion stimuli can influence behavior. In the current paper, we will focus on reactions to facial expressions as the paradigmatic case. However, our message is broader and extends to many types of emotional stimuli – thus we refer to research with emotional scenes, bodily postures, sounds, snakes, spiders, visual cliffs, etc. To preview, we will next sketch the three pathways, and later provide illustrative evidence.

## Three Pathways

**Fig. 1:**
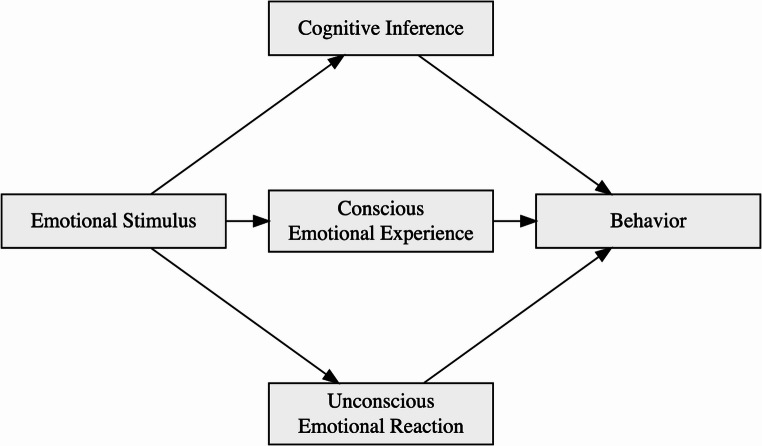
Different pathways by which emotion stimuli can influence behaviors

Figure 1 illustrates the three pathways. The first pathway by which emotional stimuli influence behavior involves purely informational or declarative processes – inferences about the stimulus meaning. In humans, we refer to these assessments as cognitive and they are discussed in the facial expression literature on social referencing and communication (e.g., Fridlund et al., 2019; Morningstar et al., 2025). The second pathway involves stimuli inducing conscious emotional experiences which then shape subsequent behaviors. The experiential route is known in the literature as “mood-as-information” (Schwarz & Clore, 2003), “feeling-as-information” (Schwarz & Clore, 2007) or “mood-as-input” (Martin, 2001) and we will hence refer to it as “feeling-as-input” pathway. The third pathway is known in the literature as “unconscious emotion” (Winkielman & Berridge, 2004). It involves stimuli (conscious or unconscious) eliciting low-level affective reactions which then influence behavior without the presence (or causal participation) of conscious emotional experience.

These pathways can be viewed through the lens of mediation models. In essence, the observation we are trying to explain is that emotional stimuli impact behavior. For example, smiling faces make people drink, enhance their risk-seeking choices, or increase donations. The mechanisms linking stimulus and behavior can be thought of as potential mediators: cognitive inference, conscious emotional experience, and unconscious emotional reaction. Note that these processes may occur together, with some playing more important role than others. However, in some cases a process (like conscious emotional experience) may not occur, or may occur but fail to play any causal role (no mediation). In the following sections, we review some illustrative studies that demonstrate the mechanisms underlying the proposed pathways. We will primarily focus on behavioral studies but will also mention some evidence from affective neuroscience.

### Pathway #1: Emotion Stimuli Influence Behavior Via Cognitive Inferences

There is a long tradition of thinking about the influence of facial expressions as relying on their communicative value (Fridlund et al., 2019; Hess & Hareli, 2017; McCullough & Reed, 2016; Morningstar et al., 2025; Parkinson, 2011; Van Kleef, 2010). It may be debated whether the underlying process in animals (Darwin, 1872) or young children should be described as cognitive inference, but it is clear that they use expressions as signals to guide behavior. For example, a caregiver’s smile communicates that it is safe to engage or approach, whereas a frown indicates the need to disengage or retreat – a phenomenon termed social referencing (Klinnert et al., 1986; Sorce et al., 1985). In adults, such inferences become more sophisticated – likely involving conscious cognitive judgments. This is evident in studies of mixed-motive situations (e.g., negotiation and bargaining, cf. Van Kleef et al., 2010). For example, in the context of negotiations, observing an opponent’s happy (or angry) expression may encourage players to continue or discontinue their current strategy (Cacioppo & Gardner, 1999; Fischer & Roseman, 2007), influencing concessions (Van Kleef et al., 2004a) and selection of tactics (van Dijk et al., 2008). The specific effects of expressions vary because perceivers use them to read the minds of their partners and opponents (de Melo et al., 2014).

The informational influence of facial expression might co-occur with other impacts. A teammate’s smile can not only signal cooperation, but also induce shared feelings of happiness, and trigger facial mimicry, such as smiling to a smile (Arnold & Winkielman, 2019). A key feature of informational effects is their contextual flexibility – they can reverse if the situational meaning of expression changes (Hess & Hareli, 2017; Lanzetta & Englis, 1989). For example, in one study, participants played a dice game with an android, who can generate human-like facial displays (Hofree et al., 2018). After each trial, the outcome of a dice throw was communicated by the android either through a facial expression or a text message. Before the game began, participants were randomly assigned to either a cooperative condition, in which the android was framed as a teammate, or a competitive condition, in which the android was framed as an opponent. As a result, the android’s facial expressions communicated distinct signals depending on the context – the teammate’s smile showed a win and a frown signaled a loss; in contrast, the opponent’s smile showed a loss and a frown signaled a win. Facial EMG analyses found that participants’ reactions were fully controlled by the information value signaled by the android’s facial expressions. In particular, participants smiled more when they won, regardless of whether this outcome was signaled by a teammate’s smile or an opponent’s frown. Conversely, participants frowned more when they lost, independent of how the outcome was signaled – with a smile or with a frown. An additional control condition also showed that communicating the outcome with a text script has similar (though weaker) effects as with facial expression. Overall, these findings suggest that participants’ behavioral reactions are largely driven by informational value of facial expressions (Hofree et al., 2018). Similar flips in the impact of expressions on participants’ motor reactions have been observed with status related variables, such as power and social rank (Carr et al., 2014).

#### Neuroscience Evidence

Interestingly, the work on neural mechanisms underlying informational impacts of facial expressions suggests involvement of cortical structures like the superior temporal sulcus and prefrontal cortex (Blair, 2003), as well as networks underpinning theory-of-mind inferences, such as TPJ (Koster-Hale & Saxe, 2013; Singer, 2009). After all, when we see an expression, we often ask “what does it really mean?” essentially inquiring about displayers’ intentions (Altschuler et al., 2021; Hess & Hareli, 2017; Van Kleef et al., 2004b).

### Pathway #2: Emotion Stimuli Influence Behavior Via Conscious Emotional Experience

Everyday life and research suggest that conscious emotional feelings guide a variety of behaviors, including complex decisions (see Keltner et al., 1993; Lerner et al., 2015; Loewenstein et al., 2001; Slovic et al., 2002; Winkielman et al., 2007; van ’t Wout et al., 2006). Facial expressions can be a powerful source of such feelings. Popular culture reflects the intuition that a smile can lift our mood and make us act generously (e.g., songs in Public Domain Music, 2024). This intuition is confirmed by research showing the impact of loan requester’s smile on positive feelings and on actual donations (Genevsky & Knutson, 2015). By contrast, a disgusted face can make us feel nauseous (Wicker et al., 2003). That feeling may stop us from eating and, more broadly, from pursuing new things, including taking risks (Sparks et al., 2018).

However, a key question in this research is whether conscious feelings actually play a causal role in behavior. After all, the underlying mechanisms proposed in this research often involve (i) spreading semantic activation, where affect induction automatically primes associated concepts (Forgas, 1995; Johnson & Tversky, 1983) or (ii) cognitive appraisals, where the influence is carried via activated cognitive dimensions, such as controllability (Lerner et al., 2015). So, is there evidence that conscious feelings actually matter?

Early empirical suggestions for the causal role of feelings come from classic studies reporting that people mistakenly link unrelated targets to feelings of arousal triggered by incidental drugs (Schachter & Singer, 1962; Reisenzein, 1983) or a suspension bridge (Dutton & Aron, 1974). Schwarz and Clore (1983) found that participants reported lower life satisfaction on rainy days compared to sunny days, unless they correctly attributed their mood to the weather. These examples illustrate that feelings can “carry over” from one situation to another, influencing even unrelated behaviors. Importantly, when people believe that the current feeling is irrelevant to the decision at hand, its impact on judgment disappears (Schwarz & Clore, 1983; see also Zanna & Cooper, 1974 for an early demonstration). This pattern is hard to explain by approaches that purely appeal to cognitive factors, such as spreading semantic activation or transfer of appraisals. After all, learning that a feeling is irrelevant does not by itself reduce semantic activation. To test the feeling-as-input perspective, one study induced participants’ anger by asking them to read a fictional story and describe a similar life experience (Srivastava et al., 2008). In the “warning” condition, participants were explicitly told that their anger stemmed from the previous task and warned not to let it influence their subsequent decisions. In the control condition, no such warning was given. When presented with either a fair offer ($5 out of $10) or an unfair offer ($1 out of $10), those in the no warning condition showed significantly higher rejection rates than those in the warning condition. This finding suggests that, in line with the feeling-as-input hypothesis, anger can drive rejection of unfair offers – unless people recognize their feelings are irrelevant (Srivastava et al., 2008).

Similar effects have been reported with facial expressions. In one study, the facial expression of the proposer influenced a responder’s acceptance or rejection of the offer in an ultimatum game (Mussel et al., 2013). The game featured a range of offers from extremely unfair to favorable. The proposer’s facial expressions were manipulated to show either happy, neutral, or angry expressions. Results showed that the acceptance rates were higher when the proposer was smiling as opposed to neutral, and lower when the proposer was frowning compared to neutral. Note that this study did not measure whether these decisions were driven by changes in the responder’s subjective feelings or situation appraisals (see also Pietroni et al., 2021). However, other studies using similar paradigms did measure subjective experiences and reported that faces can indeed induce feelings, which subsequently influence decisions. For example, Howard and Gengler (2001) found that mimicking a partner’s happy facial expression in dyadic interactions enhanced positive feelings, which led to more favorable judgments of a novel product. Genevsky and Knutson (2015) reported that a loan requester’s smile elicited more positive affect and increased funding rates. These results align with a meta-analysis by Joseph et al. (2020), which created a database with over 500 studies to test what types of emotional stimuli are most effective at affect induction. The meta-analysis suggests that facial expressions are effective at eliciting changes in mood (effect size = 2.06), comparable to other emotional stimuli. Similarly, in his review, Parkinson (2011) concluded that facial expressions can elicit mood contagion. Note, however, that some studies also report that facial expressions exert either weaker or at least different type of influence than emotional scenes on physiological measures of affect (Alpers et al., 2011; Mavratzakis et al., 2016; Wangelin et al., 2012). This suggests that sometimes facial expressions may influence behavior through other pathways than non-facial stimuli.

#### Neuroscience Evidence

Over the last 30 years, the studies on neural basis of emotional experience have consistently pointed to a wide network of activations, spanning both cortical and subcortical structures (Critchley & Garfinkel, 2017; Craig, 2009; Damasio et al., 2000; Gogolla, 2017; Gündem et al., 2022; Lindquist et al., 2016; Singer, 2009). Unfortunately, much of this evidence is correlational, leaving the neural basis of conscious experience a hotly debated topic with proposals favoring (LeDoux & Brown, 2017) and opposing (Panksepp, 2001) linking emotional experience to cortical networks. Furthermore, many of these studies do not involve any behavior, focusing only on neural reactions to various emotion induction procedures such as autobiographical recall procedures, scenarios, music, images, or movies.

In studies attempting to establish neural links between emotional stimuli and actual behavior, most research comes from decision-making, with classic studies pointing out the role of the ventro-medial prefrontal cortex (Bechara et al., 1997; Damasio, 1994) and orbitofrontal cortex (Rolls, 2023). Research using gambles also shows that insula activity correlates with subjective feelings of negative arousal and behavioral measures of risk aversion (Kuhnen & Knutson, 2005; Paulus et al., 2003), whereas nucleus accumbens and medial prefrontal cortex correlate with feelings of positive arousal and behavioral risk seeking (Knutson et al., 2008; Mortazavi et al., 2025).

With regards to facial expressions and subjective feelings, the neural evidence is surprisingly scant – probably because most neuroscientists study networks associated with recognition of emotional expression, rather than people’s subjective feelings in reactions to them (Adolphs, 2002; Blair, 2003, Pessoa & Adolphs, 2010). One notable exception is the aforementioned study on smiles and donations by Genevsky and Knutson (2015). It reported that requesters’ joyful faces robustly enhanced donors’ activity in nucleus accumbens and self-reported feelings of positive arousal. Using both neural and feeling reactions to faces allowed the researchers to successfully predict donation behavior (requester’s funding rate) on both individual and populational scale. Still, many discovered mechanisms are compatible with the idea of unconscious emotion, as we discuss next.

### Pathway #3: Emotion Stimuli Influence Behavior Via Unconscious Emotional Reactions

Several lines of research challenge the idea that conscious emotional experience is always elicited by affective stimuli, or, if elicited, plays a causal role in behavioral changes. Before we go deeper into the question of subjective experience, it is worth noting that some emotional stimuli (angry faces, snakes, spiders) are unique: they might be highly practiced, evolutionarily prepared, and individually or socially important (Öhman et al., 2000). Accordingly, it should not be surprising that they work in different ways from other emotion stimuli. Behavioral studies show biases in perception and learning of such stimuli (LoBue et al., 2010). Physiological studies comparing the impact of facial expressions versus emotional scenes report that faces elicit weaker reactions on measures of arousal (skin conductance, heart rate) than scenes but are equally or more effective in triggering orienting reactions and motor responses (Alpers et al., 2011; Mavratzakis et al., 2016; Wangelin et al., 2012). We will address neural mechanisms shortly.

Research on affective influence found that facial expressions, even presented very briefly, can impact preference judgments (Murphy & Zajonc, 1993; Niedenthal, 1990; Winkielman et al., 1997), basic approach-avoidance tendencies (Marsh et al., 2005; Seidel et al., 2010), loss processing (Schulreich & Gerhardt, 2016), risky decisions in a monetary gamble (Winkielman et al., 2022), and consumption behavior (Winkielman et al., 2005; Winkielman et al., 2018). Critically, in some of these studies, researchers explicitly probed subjective experience but found that people reported no changes in feelings (Winkielman et al., 2005). Furthermore, unlike in studies with conscious moods (e.g., Schwarz & Clore, 1983), providing an opportunity to misattribute (set aside) feelings does not alter the pattern of affective influence (Winkielman et al., 1997). Similarly, encouraging participants to use their feeling reactions to briefly flashed faces as a guide in a detection task did not improve their performance (Bornemann et al., 2012).

A skeptical reader may wonder if such “unfelt” influences are genuinely affective. However, emotional faces, even presented briefly, are successful in triggering motor, pupillary, electrodermal, and cardiovascular responses (Bornemann et al., 2012; Dimberg et al., 2000; Lapate et al., 2014; Prochazkova & Kret, 2017; Tamietto et al., 2009). Importantly, such physiological reactions are not merely reflexive, but flexibly tuned by the observer’s emotional state (Moody et al., 2007). Other studies have found that angry facial expressions increase behavioral avoidance responses compared to happy facial expressions (Averbeck & Duchaine, 2009), and that this pattern is modulated by increased skin conductance responses (SCRs) in healthy and socially anxious participants (Pittig et al., 2014). Again, it is important to highlight that in some studies participants who showed clear physiological (e.g., facial muscle) responses to briefly presented facial expressions were unable to access their own responses as “feelings”, even though the stimuli biased their risky decisions (Bornemann et al., 2012; Rohr et al., 2025). In sum, such results indicate that facial expressions can influence behavior through a genuine affective pathway, even in conditions of minimal conscious processing. We acknowledge though that strong claims of unconsciousness, in terms of both stimulus perception and the affective state, require careful assessments and replications (Stockart et al., 2025).

#### Neuroscientific Evidence

The best neural candidate for implicit affective processing is the amygdala. It responds to stimuli such as snakes (Van Le et al., 2013), spiders (Björkstrand et al., 2020), and of course, facial expressions. In fact, patients with amygdala lesions struggle to recognize fear in facial expressions (Adolphs et al., 1994), and healthy participants show heightened amygdala activation to fearful faces, even when only the white parts of the eyes were presented very briefly (Whalen et al., 2004). The amygdala’s role extends beyond fear to surprise expressions (Kim et al., 2003; for a review, see Whalen et al., 2017). Importantly, neuroscience evidence highlights that even in humans, the operation of subcortical structures, such as the amygdala, may be dissociated from conscious experience. Inman et al. (2020) demonstrated that direct stimulation of the amygdala led to physiological changes associated with emotions, such as increased heart rate, respiration, and heightened skin conductance. However, among the eight participants showing those changes, only one reported experiencing conscious fear. This dissociation between the role of amygdala in organizing valenced responses and generating conscious feeling is further highlighted by a patient S.M. who lacks amygdala, fails to show amygdala-related perceptual and learning effects, shows many “fearless behaviors”, yet can experience feelings of genuine panic (Feinstein et al., 2013).

#### Present but Causally Inert Conscious Feelings

The research just described on S.M. (Feinstein et al., 2013) and on the effects of amygdala stimulation (Inman et al., 2020) highlight that the mechanisms underlying conscious feelings can separate from mechanisms of basic affective reactions. This raises an interesting scenario (a subset of pathway 3) where a stimulus elicits conscious feelings, but they are not causally responsible for the behavior. By analogy, this scenario is an affective version of the famous Libet’s et al., (1983) argument that conscious feelings of volition can accompany motor action but do not causally drive it (cf. Desmurget & Sirigu, 2009; Dominik et al., 2024). This scenario also recalls the famous claim attributed to William James (1894/1994) that conscious feelings of emotion follow, rather than precede, behavior (but see Ellsworth, 1994 for more cautious interpretation of James’ position).

Is there evidence for a dissociation between “affective feelings” and “affective influence” in paradigms involving sophisticated behavior, such decision-making? Bechara and colleagues (1997) famously argued that affective associations (somatic markers) drive gambling decisions independently (and even prior) to such markers becoming subjectively represented as “conscious hunches”. In other words, basic affective processing can sometimes play a double causal role: (i) driving the decision and (ii) causing the conscious feeling. The key point is that the conscious feeling never (or only much later) becomes a part of the causal process of shaping the relevant decision.

A more recent study by Shaham and Aviezer (2022) provides more direct evidence for the dissociation between the impact of stimuli on emotional experience and behavior. Participants rated their subjective feelings upon seeing a set of happy and angry bodily postures. Participants were repeatedly exposed to these bodily postures, which were randomly paired with either a purple or an orange background. Through three habituation blocks, participants were instructed to frown at one of the background colors. Results showed that participants consistently frowned faster in compatible trials (i.e., when the background color corresponded to angry bodily postures) compared to incompatible trials, with no habituation over blocks. In contrast, emotional experience ratings showed a habituation effect – happy postures evoked significantly less pleasure in the final block compared to earlier blocks. Thus, the pattern of emotional experiences did not correlate with the pattern of behavioral changes. This finding provides one example where, despite emotional stimuli eliciting emotional experiences, the emotional experiences do not drive behavioral changes.

Turning to facial expressions, there are several studies on mimicry that suggest similar dissociations. Specifically, in scenarios where facial expressions evoke both mimicry and subjective feelings (i.e., emotional contagion), both effects are often causally unrelated (Blairy et al., 1999). For instance, Hess and Blairy (2001) demonstrated that observing happy or sad facial expressions led to both facial mimicry and emotional experiences. However, the intensity of these emotional experiences did not correlate with the magnitude of mimicry responses. For instance, when participants viewed happy expressions, the intensity of cheerfulness was not correlated with magnitude of increased activation of the Orbicularis oculi (a muscle associated with smiling) or decreased activation of the Corrugator supercilii. This dissociation suggests that while facial expressions can induce both mimicry and emotional experiences, emotional experience may not cause mimicry behaviors.

### Is Valence Out there? Emotional Processes Influence Intrinsic Qualities of Objects

If not via cold semantic processes or by eliciting conscious feelings, how can emotion stimuli drive behavior? This is a challenging question, especially when one considers complex behaviors, such as consumption choices, financial decision-making, or judgments of social impressions. As just discussed, one possibility runs via front-end biases in perceptual, attentional, memory and motor processes. All judgments, simple or complex, require some encoding of the target (properties of a novel drink, gains and losses of a gamble, etc.). So, these front-end processes could change how the target stimulus appears to the perceiver.

One perspective suggests that low-level affective processes do not change any emotional aspects of conscious experience and simply influences target’s perceptual salience or decision weight. This could have downstream consequences on choice by biasing evidence accumulation (Smith & Krajbich, 2019). Similarly, low-level affect changes allocation of attention to congruent options, as in studies where attention is drawn to previously rewarded features (Bennett et al., 2023). In short, unconscious affective processes can do their job of steering the individual towards different choices via control of basic non-affective processes, like perception and attention.

An alternative perspective, sometimes referred to as “affective realism”, suggests that emotion stimuli may not trigger distinct feelings but change the consciously perceived target valence (Arnaud, 2023; Barrett & Bar, 2009; Cleeremans & Tallon-Baudry, 2022). This proposal fits with a venerable tradition of thinking about emotions as modifiers of intrinsic quality of objects – a property that makes emotions so subjectively compelling. Zajonc (1980) wrote that nearly all objects of conscious perception (even a simple house) appear as valenced or at least “micro-valenced”. Frijda (2007) discussed how a baby’s charm appears to parents as an intrinsic quality, as if the baby objectively possesses it, rather than a “misattribution” of parents’ warm feelings. This perspective aligns with research showing that facial expressions can alter the perceived qualities of subsequent objects. In the 2005 “Kool-aid” study by Winkielman and colleagues, participants exposed to briefly presented happy faces not only consumed the beverage more, but also rated the beverage more favorably compared to those exposed to angry faces, all while denying any change in feelings. Though this study is often used to make a point that affect is entirely unconscious, the findings can be reinterpreted as suggesting that incidental affective faces changed the *consciously perceived* intrinsic affective quality of the drinks. Importantly, this perspective agrees on the key point -- the absence of distinct “feelings” available to verbal reports, endorsement, feeling misattribution, transfer, correction, or simply setting aside (see Winkielman et al., 1997). In other words, the process of implicit affective influence could be described as a form of low-level “affective misattribution” (Loersch & Payne, 2011), but it is not a misattribution of distinct conscious feelings, as articulated by the mood-as-information (Schwarz & Clore, 2003), feeling-as-information (Schwarz & Clore, 2007), or mood-as-input perspectives (Martin, 2001).

The argument that non-experiential (not feeling-based) affective influence involves changes in perception of target’s valence is consistent with additional evidence from psychology. Studies using Continuous Flash Suppression (CFS) found that a neutral face could be perceived as happier or sadder, depending on whether it was preceded by a suppressed happy or sad expression (Siegel et al., 2018). A similar CFS paradigm showed that neutral faces paired with suppressed smiling faces were rated with more positive traits (e.g., more trustworthy) than with suppressed scowling faces (Anderson et al., 2012; Wormwood et al., 2019). Importantly, changes in perception of face emotionality cannot be always explained by a mechanism of transfer (mistaken binding) of perceptual features from the incidental stimulus to the target. For example, an affective manipulation such as mere-exposure (repetition without any change in target features) improves how positively the faces look to participants, as tested with rigorous perceptual measures (Carr et al., 2017). In short, it appears that basic affective processes can change perception of target valence – which is perceived as intrinsic to the target – without involving a distinct mood-like conscious feeling.

Turning to neuroscience, illustrative examples come from work showing that the amygdala is particularly sensitive to low-spatial frequencies which contain information about fear (Critchley et al., 2000; Yang & Yeh, 2018; Vuilleumier et al., 2003; Wang et al., 2023). Several studies found that fearful facial expressions can enhance sensitivity and alter subsequent judgments of stimuli, such as Gabor patterns presented in low-spatial frequency (Bocanegra & Zeelenberg, 2009; Phelps et al., 2006). Notably, the modulation of attention and perception may be unique to negative facial expressions, particularly fearful and angry facial expressions. This is because such faces are especially potent at activating the amygdala, and in turn boosting sensory processing via its connections to the visual cortex (Anderson & Phelps, 2001; Kesler-West et al., 2001; Morris et al., 1998; Smith et al., 2005). In fact, angry and fearful facial expressions, even presented briefly, are able to modulate gaze behavior (e.g., Vetter et al., 2019).

All these considerations suggest concrete mechanisms by which emotional stimuli, including faces, can influence decisions via front-end processes, without the presence or causal involvement of distinct feelings. Clearly, further work is needed on the challenging question of whether these effects involve changes in consciously perceived target valence, or operate even deeper, without any changes in affective aspects of conscious perception. Note that the latter possibility better connects human and animal research, with many documented affective influences on decision-making in mammals (Harding et al., 2004) and even insects (Solvi et al., 2016), where conscious processes are unlikely to be the right explanatory mechanism.

## Summary, Implications and Conclusions

In summary, we examined the influence of emotional stimuli on behavior, with special emphasis on facial expressions. We proposed three pathways. The first pathway involves facial expressions serving as informational signals which influence behavior via inferences. Some of these inferences may be spontaneous, but the literature also shows them to be flexible, context dependent, sensitive to considerations of strategic communication, and thus often conscious. The second pathway covers scenarios where facial expressions are inducers of feelings. This change in subjective experience influences behavior via the feeling-as-input mechanisms, as revealed by people’s ability to strategically use their feelings, correct for their influence, or set them aside. The third pathway encompasses scenarios where facial expressions influence behavior without the presence or the causal involvement of emotional feelings. Here facial expressions may trigger low-level affective reactions that bias perception, attention, memory, motor and motivational processes. As noted earlier, the key element of the pathways is the presence of low-level but genuinely affective processes, which can occur without, or be dissociated from feelings.

Our proposal emphasizes that these pathways are not mutually exclusive. In most cases, emotional stimuli shape behavior via multiple mechanisms. This multiplicity allows humans to benefit from spontaneous unconscious processes as well as deliberative conscious processes that ensure flexibility in emotional responding (Cleeremans & Tallon-Baudry, 2022; Ludwig, 2025). In fact, research shows that facial expressions, especially in their dynamic form, transmit a rich and evolving hierarchy of signals (Jack et al., 2014). At the initial stages of this hierarchy, information such as detectability (e.g., sudden movement, high contrast typical of danger signals) can increase the salience of the facial stimuli, making them primarily “attention grabbers,” “perception biasers”, or “simple motor triggers”. At the later stages, facial stimuli elicit interpretation and categorization processes, influencing basic social impressions (Kaminska et al., 2020). At the final processing stages, faces serve as a basis of inferences about the displayer’s complex intentions and communicative goals (de Melo et al., 2014; Koster-Halle & Saxe, 2013; Morningstar et al., 2025). Future research should aim to clarify the specific psychological and neural mechanisms underlying these pathways and their relative roles in different forms of emotional influence. This would mean examining the hypothesized mediators simultaneously, including perception, attention, physiology, brain activation, conscious emotional experiences, attribution and cognitive judgements.

Central to future investigations is appreciating that expressions may vary widely in their ability to trigger different processes. Some specific facial expressions (or facial expression sets) may be good triggers of conscious feelings, some work best at generating limbic activation, others may be best at eliciting motor mimicry, and yet others may be best at influencing strategic decisions. It is interesting, for example, that most research on low-level attentional and perceptual biases usually involves angry or fearful faces, whereas research on avoidance responses often uses disgust stimuli. This could be due to their adaptive significance, but also general potency of negative stimuli (Joseph et al., 2020). Nevertheless, happy faces are usually good triggers of motor mimicry, perhaps due to stronger perception-action links (Arnold & Winkielman, 2019) or greater social value of happiness mimicry (Niedenthal et al., 2016). In contrast, sad faces may be efficient at triggering empathetic emotion contagion, without much contribution from mimicry (Olszanowski et al., 2019), whereas painful faces may trigger prosocial action, without necessarily inducing empathetic contagion (Summers & Lloyd, n.d.).

One important moderator is naturalness and genuineness of facial expressions. For example, studies have shown that natural, spontaneous dynamic stimuli influence emotional experience and behavioral responses differently from “fake”, posed, or deliberate ones (for a review, see Dawel et al., 2025). One way such natural stimuli may be able to better drive bodily response is through organically acquired perception-action links. Consistent with this idea, people show stronger imitation effects when they observe movements (facial or gestural) that resemble natural human dynamics (e.g., Hofree et al., 2014, 2015). Future work on developing databases of facial expressions could measure and report the relative ability of different datasets or different faces to elicit various processes. This would complement and extend work that uses classic ratings of social impressions (Ma et al., 2015). As such, we hope that our proposal informs both basic and applied research on facial expressions.

## Additional Information

### Data Availability

No datasets were generated or analysed during the current study.
